# Selection of Effective Temperature for Thermal Regeneration of Spent Alkali-Phenolic Binder Moulding Sand

**DOI:** 10.3390/ma16247629

**Published:** 2023-12-13

**Authors:** Mariusz Łucarz

**Affiliations:** Faculty of Foundry Engineering, AGH University of Science and Technology, Reymonta 23 St., 30-059 Krakow, Poland; eumar@agh.edu.pl; Tel.: +48-12-617-27-21

**Keywords:** alkali-phenolic resin, thermogravimetric analysis, spent moulding sand, thermal regeneration, process economics

## Abstract

This article presents the results of research on alkali-phenolic binders used in moulding sands. The results of the presented experiments are part of a search for the optimum conditions to implement the thermal regeneration of spent alkali-phenolic binder moulding sands. The cured binders were subjected to thermogravimetric analysis in aerobic and anaerobic atmospheres. On the basis of the resin decomposition curves, the integral polymer decomposition temperature was determined, and the required thermal regeneration temperature for the alkali-phenolic binder moulding sands that were used was determined according to a specific procedure. The spent moulding sand was subjected to roasting procedures at different temperatures in order to confirm the necessary regeneration temperatures. The effects of the procedures that were carried out were evaluated by means of weight losses and ignition losses, microscopic pictures were taken, and using scanning microscopy, an analysis of the chemical composition in the micro areas on the surfaces of the matrix grains was carried out using scanning microscopy. The indicators for the comparisons between the individual binders were also calculated from the mass losses. The research and analysis that were carried out made it possible to indicate the required temperature for the thermal regeneration of the spent alkali-phenolic binder moulding sand to reduce the involved energy expenditure. The factors that can determine the successful implementation of the process and the obtaining of the best possible grain matrix for reuse were also indicated.

## 1. Introduction

For ecological reasons, modern foundry engineering is looking for moulds and core sand binders that have the lowest environmental impacts. Methods are being developed for the binding of moulding and core sand so that the largest possible amount of a grain matrix can also be easily recovered after the production process. These efforts are dictated by the depletion of deposits of good foundry sands and the increasing cost of storing used moulding sand in landfills [1,2,3]. Comprehensive knowledge of the properties of the organic binders that are used is important for reducing the amount of waste that is sent to landfills while also creating the least energy-intensive process for recovering the grain matrixes left in foundries.

The regeneration of spent moulding sand is a way that allows a grain matrix to be recovered and used in casting production on a multicycle basis [3]. Attempts have also been made to use spent moulding sand in other industries such as road construction and the construction industry [4,5,6,7,8]. However, the best way to manage used moulding sand is reusing it in the foundry for the same bonding material technology.

There are three main regeneration methods: wet, dry (mechanical or pneumatic), and thermal [2,3]. Each of these methods has its own advantages, and each has its own disadvantages and areas of application. In [9,10,11], the authors present grain matrix recovery methods using the wet method. The authors of [12,13,14,15,16,17,18,19,20,21,22,23,24,25,26,27,28,29,30,31,32,33,34,35] have presented various solutions for mechanical reclamation. However, refs. [36,37,38,39,40,41,42,43,44,45,46] have presented issues that are related to thermal regeneration.

A binder that is used in a moulding compound must guarantee adequate resistance when in contact with a liquid casting alloy over a certain period of time while at the same time exhibiting adequate interaction with the changing properties of the casting alloy. An alkali-phenolic resin is an adhesive that meets these conditions. Its main advantage is its two-stage bonding. Initially, sufficient strength is achieved at an ambient temperature to carry out the required casting mould operations, while the essential bonding process takes place from the temperature of the liquid casting alloy. The alkali-phenolic resin exhibits thermoplastic properties during the first period of exposure to temperature, compensating for the thermal expansion of the sand during the pouring of the liquid alloy into the mould, this eliminates cracking and the possible outflow of liquid alloy. This property of the binder simultaneously affects the high dimensional accuracy and ability to resist penetration by liquid casting alloy [47,48].

When in combination with a grain matrix, alkali-phenolic resins generally result in the lower tensile strength of a moulding compound than other organic resins [49]. However, the tensile strength is sufficient for the accepted requirements of the core and mould technology in most applications while maintaining satisfactory surface quality of the castings [50]. An advantage of the alkali-phenolic binder is the significantly lower total amount of gaseous substances that are released during the pouring of the liquid casting alloy into the mould than with other organic resins [51]. At the same time, its advantages include low odour, virtually no smoke, ease of removal, good finish, low veining, minimal erosion, and very good hot strength [49].

Particularly in the production of steel castings, alkali-phenolic resin moulding sand preparation technology has become the standard [52] and has been accepted as the most convenient and environmentally protective. The advantages of the alkali-phenolic process relate to improved casting quality and the reduced of the costs that are associated with casting cleaning. In [53], the authors stated that the alkali-phenolic system led to higher environmental acceptability. Gaseous emissions from an alkali-phenolic system are two to five times lower than for a furan resin moulding compound (without burning).

The alkali-phenol process is a binary bonding system that consists of an aqueous reactor and liquid ester as a reaction partner. The moulding compound that is produced in this process uses alkali-phenolic resins that have been cured with an organic (alcohol-based) ester. The phenolic resins that are used in the system are prepared by reacting a 30–55% formaldehyde solution with a smaller amount of phenol (using a strong alkaline catalyst such as sodium or potassium hydroxide) at temperatures below 110 °C (pH > 7) [49]. Foundries considered the difference between the use of NaOH and KOH in the preparation of alkali-phenolic resins. In line with the opinion presented in [54], the author believed that the achievement of the required strength by a particular binder was dependent on the composition and parameters of the process rather than the type of alkali compounds used. The author also stated that the development of a suitable procedure in a given foundry requires a great deal of skill.

However, the type of principle used is important in terms of the planned regeneration method. In a series of tests that used different regeneration methods and equipment, it was found that the weaker strength of the moulding compound on the regenerate matrix was due to the existing small number of inorganic compounds on the surface of the sand. When recovering the grain matrix after the alkali-phenolic process by mechanical methods, it must be taken into account that the alkali metals that are in the composition of phenolic resins can react with the surface of the grain matrix during setting. The alkali compounds can change the surface of the chemical reaction and remain on the grain matrix [49]. The formed alkali salts have a greater effect on the grain matrix after thermal regeneration than they do after mechanical regeneration. When reacting with quartz sand, they form a silica glaze. The silicates that are formed as the result of sintering deteriorate the performance of the moulding sand that is made on the grain matrix after the regeneration process. In addition, the sodium and potassium salts that accumulate on the surface of the regenerated grain matrix (especially in the irregularities) can reduce the tensile strength. To prevent this, special additives (0.6–1.0%) are added to the spent matrix just before the thermal regeneration process. The mineral additives used minimize the sintering temperature of the granules by combining with alkali metals and are removed during the pneumatic grading process as fine particles [54].

The basic composition for preparing a quartz matrix moulding compound with an alkali-phenolic binder involves the addition of one to two parts of alkali phenolic resin (resol) by weight, followed by adding about 18 to 25% aliphatic ester to the resin. In the case of chromite and olivine sand, more resin is added (about two parts by weight), and the ester is added at a resin/hardener ratio of 5/1. The wide range of hardeners allows for the production of moulding compounds with different curing times, which is one of the most important parameters of the alkali-phenolic resin-bonded moulding compound. The curing time of the moulding compound can be adjusted by the type of ester that is used (ranging from 5 to 30 min) [55,56,57,58].

According to the authors of [59], the increasing environmental demands in and around the foundry should direct efforts towards developing the optimal properties of the moulding compounds for the production of castings. This is an important issue for the European foundry industry, which sees a solution to this situation in the use of the alkali-phenolic process [59]. Currently, the commonly used furan resin in steel foundries is being replaced by an alkali-phenolic resin for environmental and economic reasons [54]. However, this is a difficult process that requires the complete replacement of the circulating acidic regenerate with a new grain matrix that will be alkaline in nature. The results of the tests on the moulding of compounds when using several alkali-phenolic resins (produced by different manufacturers and using different grain matrices) were presented by the authors of [60]. On the basis of comparative studies (adopting the criterion of the highest moulding sand strength), it was concluded that a suitable alkali-phenolic resin could be identified, which is predisposed as a substitute for furan resin.

In the case of moulding sand with a binder that contains organic components, thermal regeneration is the method that allows for the purest matrix to be obtained. At the same time, it is the method with the highest grain matrix yield [1] (unlike the mechanical method, which causes the successive abrasion of the grain surface) [61]. Due to the considerable energy intensity of the process, however, it seems advisable to seek solutions for reducing the costs that are associated with the implementation of thermal regeneration. The costs of the mechanical regeneration process and the promotion of this method for the recovery of waste foundry sand (WFS) have been presented in publications [1,16].

Thermogravimetric analysis is such a study that allows for the properties of the binder that is used to prepare a moulding or core sand to be known (particularly in view of its degradation and destruction under certain temperature conditions), thus creating guidelines for the regeneration of various organic binders. An indicator of the suitability of binders for foundry applications is their thermostability. Various methods for determining the thermostability of polymers have been presented in [1,62]. Of particular note is the determination of the integral procedural decomposition temperature (ipdt) index, which was developed by Doyle (1961). Based on a study of 54 polymers, he created an index to compare the stability of different polymers with each other. A notable feature of this method is that none of the previously proposed methods took the slope or shape of the TG curve into account. These features of the TG curve are closely related to the course of the polymer degradation process. Thermal resistance tests should be conducted in an inert gas atmosphere to avoid the influence of the atmosphere on the degradation process. The ipdt index refers to the TG curves that are recorded within a fixed temperature range of 25–900 °C.

## 2. Materials and Methods

### 2.1. Materials

In this study, three alkali-phenolic resins from different manufacturers were used, along with their respective hardeners. The individual resins were combined with their hardeners in order to perform a thermogravimetric analysis of the binder. Table 1 summarises the resins that were used and their respective hardeners, as well as the amounts of the hardeners that were added to the resins.

Based on the binders that are presented above, the moulding compounds were prepared with the following composition:

- SIBELCO grain matrix with parameters (average arithmetic grain size—d_a_ = 0.29 mm; main fraction—85%)—100 parts by weight;
- Resin in the amount of 1.2% relative to the grain matrix;
- Hardener in amount of 25% relative to the resin.

Thus, three different masses were obtained and designated according to the binders that were used: moulding compounds MSA1, MSA2, and MSA3.

### 2.2. Methods

A thermal analysis test was conducted to evaluate the mass changes of the alkali-phenolic binders. The test was carried out using a TA Instruments SDT Q600 thermal analyser (DSC/TGA) (New Castle, DE, USA). The mass of the resin samples subjected to thermal analysis was about 20 mg, which was poured into alumina crucibles. The oven was heated at 10 °C/min to a temperature of 1000 °C, at which point the organic compounds should be completely degraded and destroyed.

To test the effect of temperature on the moulding compounds, portions of the compound with three alkali-phenolic binders were made in the same way and according to the same procedure. A constant amount of resin and hardener was added to the fresh grain matrix in each case. All of the masses were prepared in a Vogel & Schemmann Maschinen GMBH paddle mixer (type Labor Mischka 00GF/79) (Hagen, Germany). The hardener was first added to the respective grain matrix and mixed for 60 s, followed by the resin and mixed again for 60 s.

An important parameter for assessing the effectiveness of the thermal regeneration of the used moulding sand is the roasting loss. The bulk materials were subjected to roasting in an SNOL 8.2/1100 resistance furnace Alchem (Poznań, Poland). Whenever a reference is made to the loss of ignition (LOI), the mean value of two parallel determinations that are carried out on 30 g samples weighed out in quartz crucibles should be taken. The determination was carried out for the following conditions: heating and cooling with an oven; annealing temperature—1000 °C; and annealing time—2 h. It should be emphasised that such an operation makes it possible to determine the greatest amount of organic material that has degraded and destroyed (i.e., burnt away in an oxygen atmosphere). On the contrary, any determinations at temperatures that are lower than 1000 °C and at times that are less than 2 h should be considered as weight losses (WLs).

The loss on ignition (LOI) or weight loss (WL) is determined by Formula (1), in which m_i_ is the initial sample mass (in g), m_f_ is the final sample mass (in g), and m is the sample mass that is taken for analysis (in g).

(1) $$LOI=\frac{m_{i}-m_{f}}{m}\cdot 100\%$$

Determining the weight loss (WL) and loss on ignition (LOI) makes it possible to determine several comparative parameters necessary for estimating the thermal resistance of the organic binder that is used, on the one hand, and the effectiveness of the thermal regeneration process, on the other.

An indicator (W_ETR_) that can be proposed to evaluate the effectiveness of the thermal regeneration process is a comparison of the weight loss at a given temperature (WL_T_) and a given annealing time to the total amount of binder that is determined by the loss on ignition (LOI). This quantity can be written as Equation (2):

(2) $$W_{ETR}=\frac{{WL}_{T}}{LOI}\cdot 100\%$$

Determining the mass change as a function of temperature can also be used to determine the thermal resistance (R_T_) of the moulding compound for a given operating time, which can be written as Equation (5):

(3) $$R_{T}=\left(1-\frac{{WL}_{T}}{LOI}\right)\cdot 100\%$$

The relationships that are presented above make it possible to assess the effect of a temperature change in the breakdown of a casting mould on the binder in a spent moulding or core mass. However, it should be noted that mass loss and ignition losses are determined when air is freely available to the thermally treated material. The heat wave of the metal that is poured into the mould cavity spreads, creating conditions of restricted access to the air. The progressive thermal decomposition of the bound casting binder (polymer) will be the source of significant quantities of gases that, when moving outside the mould, displace any air that may have accumulated from the intergrain spaces of the moulding sand.

Images of the grain matrix after heat treatment were taken using a Keyence VHX-7000 digital microscope (Keyence Ltd., HQ & Laboratories, Osaka, Japan), recording the shape of the matrix grains in order to evaluate the resulting shapes. A 4K precision microscope that captures high-resolution images of 3D objects with the DFD 2.0 algorithm was used.

The quality of the grain matrix was assessed by observing the grain surface for its shape and the presence of resin residues. This study was carried out by scanning electron microscopy (SEM) using a Tescan Mira high-resolution microscope (Brno, Czech Republic) with an FEG electron source. The topography of the sample was studied using solid-state detectors; the beam energy was 20 keV. The low vacuum mode was used for the imaging.

The surface was observed in backscattered electron contrast (BSE and BSE COMPO), with a material contrast. The chemical composition was analysed at selected points on the grain surface and in the forms of maps of the occurrence of individual elements by energy-dispersive X-ray spectroscopy (EDS) using an Ultim Max EDS detector from Oxford Instruments (Abingdon, UK). The studies were carried out in characteristic areas at a magnification of 1000×.

### 2.3. Experiment

#### 2.3.1. Determination of the Thermostability of the Resin

**Figure 1:**
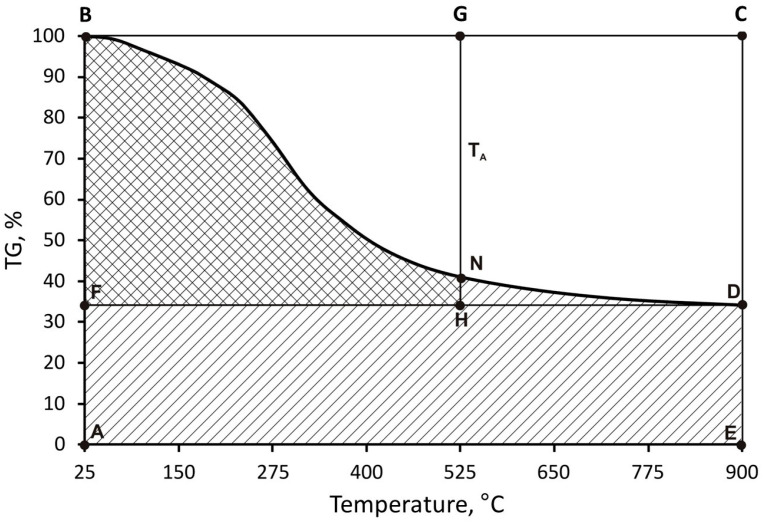
Illustration of method for calculating integral decomposition temperature.

The idea behind the procedure for determining the thermostability of the resin is essentially the calculation of the fields on the thermogravimetric analysis chart. Figure 1 graphically illustrates the method to calculate the integral temperature of the ipdt distribution.

The area of ABDE under the TG curve divided by the area of the entire ABCE rectangle gives the fraction A*, normalised to the temperature and the residual sample mass that remains after decomposition. From fraction A*, the temperature T_A_ can be obtained by multiplying it by 875 °C (the difference between 900 °C and 25 °C) and adding 25 °C according to Formula (4):

(4) $$T_{A}=875\times A^{*}+25$$

A T_A_ temperature of less than 900 °C can be taken as a temperature at which all of the gaseous products of the polymer decomposition escape. This magnitude is highly dependent on the residual mass of the sample that remains after the destruction. The calculated T_A_ temperature only serves to determine the area of the small FBGH rectangle with the double-curved HFBN area under the TG thermogram. The determined area of HFBN is divided by the area of the FBGH rectangle, obtaining another fraction K*. The Product A*K* can serve as an indicator of the thermostability of the polymers (casting resins). This index can be converted to the integral ipdt decomposition temperature according to Formula (5):

(5) $$ipdt=875\times A^{*}\times K^{*}+25$$

As presented in [1,62], this index corresponds to the temperature at which half of the gaseous products that are released during the decomposition of the polymer (binder) escape. The ipdt temperature characterises both the entire decomposition process and its rate. It is a largely reproducible quantity that is affected little by random irregularities in the thermogravimetric diagram or by systematic errors that are made during its recording. To a small extent, it also depends on slight variations in the heating rate. It is a quantity that is derived from the entire TG curve.

#### 2.3.2. Designation of the Required Regeneration Temperature

**Figure 2:**
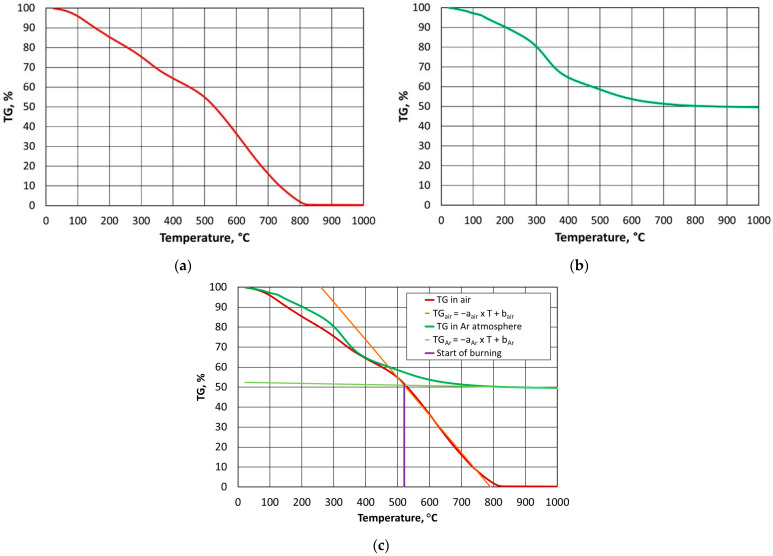
TG curves for furan binder samples: (**a**) in oxygen atmosphere; (**b**) in oxygen-free atmosphere (argon); (**c**) determination of required regeneration temperature.

In order to reduce the cost of the thermal regeneration, it is important to determine the required thermal regeneration temperature (i.e., one that is necessary to burn the spent binder and requires as little energy as possible). The author of [1] presented a suitable laboratory method for determining such a temperature for a furan binder. The procedure that was developed and described in detail in [1] for determining the required regeneration temperature for a given organic binder is based on a thermogravimetric analysis that is carried out in air and in an oxygen-free atmosphere. A characteristic feature of the obtained TG diagram (Figure 2a) for the exemplary furan binder was that, once a certain temperature was exceeded, a rectilinear section of the mass change of the tested binder sample was noticeable (a temperature range of 477 °C to 789 °C). At lower temperatures, the bonded resin degraded (a curvilinear percentage course of the mass change), while the proportional mass loss was taken as the successive combustion of the binder (which varied by less than 0.10% from 812 °C onwards). A similar relationship can be seen in Figure 2b, with thermogravimetric analysis under anaerobic conditions (argon atmosphere). As the temperature in the analyser chamber increased from a certain value, the mass of the test sample changed linearly (a temperature range of 845 °C to 1000 °C).

If we compare the data in Figure 2a to the results that are shown in Figure 2b, it can be concluded that above a certain temperature range, the breakdown of the furan binder (degradation) occurred regardless of the atmosphere in which the process was carried out. The changes in the TG values were not identical, but they followed a similar pattern. Only when above 500 °C did the atmosphere in which the test was carried out begin to play a role. In the presence of atmospheric oxygen, the sample burned (was destroyed), hence the almost proportional weight loss of the furan binder. In contrast, the second sample did not fully decompose up to a temperature of 1000 °C in the absence of oxygen in the analyser’s working space (Figure 2b); this left approximately 49.54% of the sample mass, which contained carbonised carbon.

If straight lines are drawn through the rectilinear sections of the obtained curves, their point of intersection can be taken as the minimum temperature that is required for the thermal regeneration of the binder under study. From this limiting temperature, there is the successive combustion of the residual decomposition products (carbonised carbon) that result from the degradation of the binder. An analysis of Figure 2a shows that the complete combustion process occurred at a temperature of approximately 812 °C; however, it should be noted that it was not the temperature that played the main role but the time that was required for the full combustion (oxidation) of the test binder sample.

In order to determine the minimum required regeneration temperature, a linear system, as detailed in Equation (6), was introduced, which generally take the following form [1]:

(6) $$\left\{\begin{array}{c} {TG}_{air}=a_{air}\times T+b_{air}\\ {TG}_{Ar}=a_{Ar}\times T+b_{Ar} \end{array}\right.$$

The following mathematical procedure was adopted for determining the equations. For the thermogravimetric analysis in air (TG_air_) and an argon atmosphere (TG_Ar_), the difference between the individual percentage mass losses was calculated for a constant temperature change between the following individual values (7):

(7) $$∆TG={TG}_{i}-{TG}_{i-1}$$

For the analysis that was carried out in air, the maximum change value of ΔTG_max_ was above 450 °C, which corresponded to the greatest slope of the analysed curve to the 0X axis (the greatest angle between the mass loss and the constant temperature jump). All of the percentage mass losses that were greater than half of the value of ΔTG_max_ were taken as the proportional change in the graph.

For the tests in the argon atmosphere, the minimum change value of ΔTG_min_ was above 450 °C. In this case, it was estimated that a proportional change in the thermal analysis curve would occur when the percentage losses were no greater than double the ΔTG_min_ value, which corresponded to the smallest slopes of the individual calculated values on the 0X axis.

The point of intersection of the determined linear functions should be taken as the value sought (8):

(8) $${TG}_{air}f\left(T\right)={TG}_{Ar}f\left(T\right)$$

It was decided to apply the algorithm that was prepared in this way to the alkali-phenolic binder, which also has inorganic compounds in its composition and was therefore nonflammable.

## 3. Results

**Figure 3:**
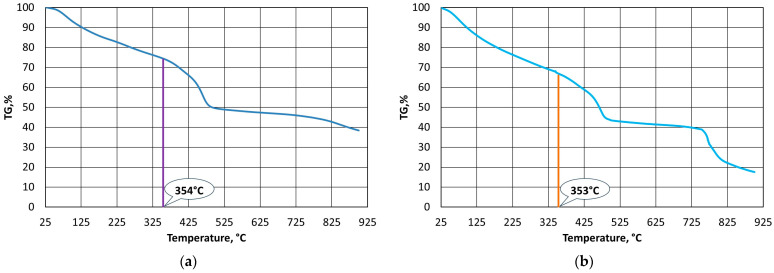
Thermogravimetric analysis of A1 binder: (**a**) in oxygen-free atmosphere; (**b**) in oxygen atmosphere.

**Figure 4:**
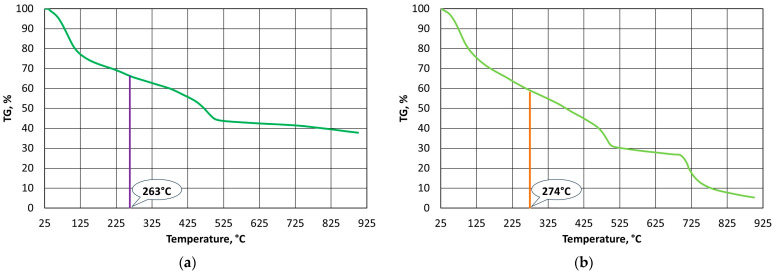
Thermogravimetric analysis of A2 binder: (**a**) in oxygen-free atmosphere; (**b**) in oxygen atmosphere.

**Figure 5:**
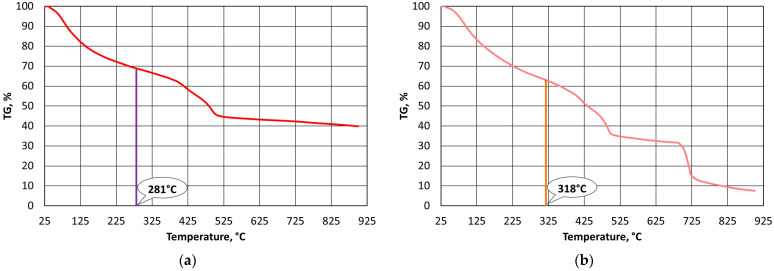
Thermogravimetric analysis of A3 binder: (**a**) in oxygen-free atmosphere; (**b**) in oxygen atmosphere.

A thermogravimetric analysis of the bound alkali-phenolic resins was performed in order to determine their thermostability. Figure 3, Figure 4 and Figure 5 show the results that were obtained for the individual resins under oxygen-free and oxygen atmospheres.

In the oxygen-free atmosphere, all of the binders degraded to the following levels: A1—38.43%; A2—37.82%; and A3—39.86%. This was in line with the previous finding. On the contrary, the samples degraded to the following levels in the oxygen atmosphere: A1—17.60%; A2—5.34%; and A3—7.54%.

Based on the methodology that is described in Section 2.3.1, the individual field values (9) were calculated according to the assumed procedure.

(9) $$Surface\;area=\sum \frac{\left(y_{i}+y_{i-1}\right)\times \left(x_{i}-x_{i-1}\right)}{2}$$

The results of the calculations for all of the analysed resins are summarised in Table 2 according to the Doyle method. The calculated values of the integral ipdt decomposition temperature are shown in Figure 3a through Figure 5a.

The procedure that was presented in [62] involved determining the thermostability of polymers in an oxygen-free atmosphere, in which the test material sample was degraded and then destroyed by pyrolysis (charring). This way of determining the thermostability of a polymer can be useful in the case of a casting mould, where conditions of limited access to oxygen from the air can be created. In the case of thermal regeneration, it is important that destruction does not merely char the casting binder but burns it. Therefore, calculations were performed under the TG curve in an oxygen atmosphere using the same procedure. To distinguish other conditions of implementation, the designations of the calculated parameters were changed. Table 3 shows the calculated fields and parameters A*_(O)_ and K*_(O)_, along with the temperature of the full volatilisation of all of the gaseous products from the polymer decomposition in an oxygen atmosphere T_A(O)_, and the ipdt_(O)_ temperature of the release of half of the gaseous products from the polymer. The integral idpt_(O)_ decomposition temperature in an oxygen atmosphere is shown in Figure 3b through Figure 5b.

The values that are collected in Table 2 and Table 3 indicated that depending on the chemical composition of the resin, different relationships were obtained between the ipdt and ipdt_(O)_ integral temperatures. From the results, it can be concluded that the more inorganic compounds in the binder (A1 resin) not burning, the higher the thermostability, and that the calculated integral decomposition temperature values in oxygen and oxygen-free atmospheres have a similar values.

**Figure 6:**
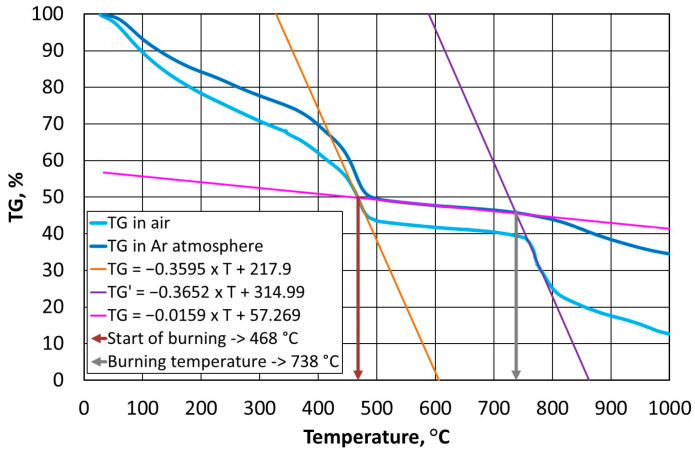
Selection of burning temperature for A1 binder.

**Figure 7:**
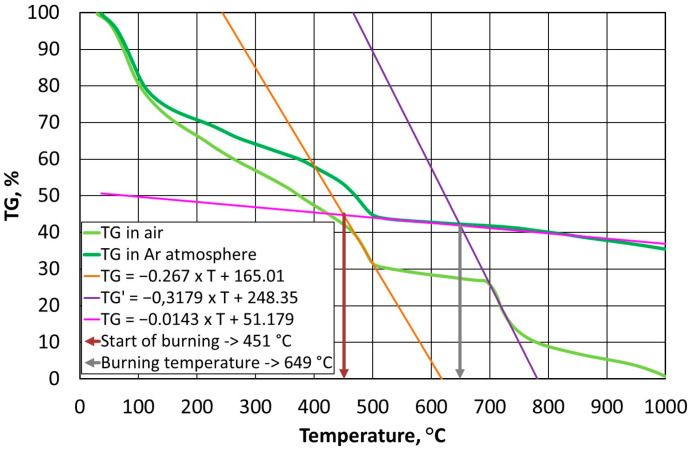
Selection of burning temperature for A2 binder.

**Figure 8:**
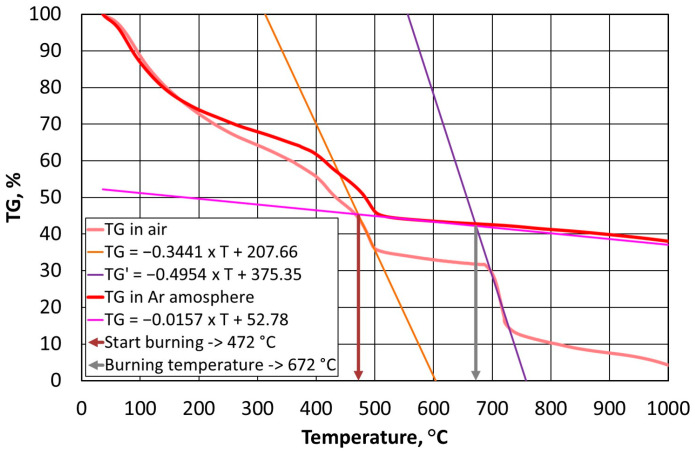
Selection of burning temperature for A3 binder.

Thermostability defines the temperature resistance of a resin, which is an important parameter from the point of view of the durability of a mould and the reproducibility of the assumed shape of a casting with great precision. On the other hand, the used grain matrix must be regenerated if we wish to keep it in circulation. By making this thermal process as energy-consuming as possible, the required regeneration temperature (i.e., the temperature that is required to burn off the resin in question) can be determined. In the case of organic resins without inorganic additives, determining this temperature is straightforward according to the methodology that is described in Section 2.3.2; however, this process becomes more complicated in the cases of alkali-phenolic resins. As can be seen in Figure 3b, Figure 4b and Figure 5b, the degradation process follows a similar course, while the destruction (combustion) part is characterised by a transition episode that is spread over three stages. Figure 6, Figure 7 and Figure 8 show how to determine the required temperature of the tested binders according to the described methodology with modifications for an additional stage of destruction of any alkali-phenolic binder.

Based on the recorded thermogravimetric analysis data for the A1 binder, the systems of Equations (10) and (11) were determined.

(10) $$\left\{\begin{array}{c} {TG}_{air}=-0.3595\times T+217.9,\;R^{2}=0.9994\\ {TG}_{Ar}=-0.0159\times T+57.269,\;R^{2}=0.9979 \end{array}\right.$$

(11) $$\left\{\begin{array}{c} {{TG}^{\prime }}_{air}=-0.3652\times T+314.99,\;R^{2}=0.9694\\ {TG}_{Ar}=-0.0159\times T+57.269,\;R^{2}=0.9979 \end{array}\right.$$

The solution to Equation (10) is the onset of the burning temperature of the binder of T_s_ = 468 °C, while from Equation (11) for A1 binder, the temperature that is needed for the burning of T_b_ = 738 °C can be determined (Figure 6).

The same procedure was followed in an identical manner as with the A1 binder, and the systems of Equations (12) and (13) were determined for the A2 binder.

(12) $$\left\{\begin{array}{c} {TG}_{air}=-0.267\times T+165.01,\;R^{2}=0.9943\\ {TG}_{Ar}=-0.0143\times T+51.179,\;R^{2}=0.9928 \end{array}\right.$$

(13) $$\left\{\begin{array}{c} {{TG}^{\prime }}_{air}=-0.3179\times T+248.35,\;R^{2}=0.9839\\ {TG}_{Ar}=-0.0143\times T+51.179,\;R^{2}=0.9928 \end{array}\right.$$

Based on Equation (12), the onset of the burning temperature of the A2 binder was determined to be T_s_ = 451 °C, while solving Equation (13) allowed the burning temperature of T_b_ = 649 °C to be determined (Figure 7).

The results of the analyses for the A3 binder are shown in Equations (14) and (15).

(14) $$\left\{\begin{array}{c} {TG}_{air}=-0.3441\times T+207.66,\;R^{2}=0.9979\\ {TG}_{Ar}=-0.0157\times T+52.78,\;R^{2}=0.9935 \end{array}\right.$$

(15) $$\left\{\begin{array}{c} {{TG}^{\prime }}_{air}=-0.634\times T+474.7,\;R^{2}=0.9929\\ {TG}_{Ar}=-0.0157\times T+52.78,\;R^{2}=0.9935 \end{array}\right.$$

Equation (14) for the A3 binder allows for the calculation of the onset temperature, which is T_s_ = 472 °C, while the solution of Equation (15) is the burning temperature of T_b_ = 672 °C (Figure 8).

Table 4 summarises the calculated values of the individual parameters that were analysed.

The research that is presented here allows for the following observations to be made. The thermogravimetric analyses in the oxygen-free atmosphere for all of the binders tested yielded similar results in terms of the amount of the remaining unburned parts of the binders. However, the percentages of the unburned portions of the sample varied more in the oxygen atmosphere, The thermogravimetric analyses of the tested binders in the oxygen-free atmosphere showed that despite the similar residual binder values that were obtained, the calculated temperatures did not depend on this residual value. The temperatures that were tested in the oxygen atmosphere developed differently; the greater the amount of inorganic binder residue (non-combustible), the higher the calculated ipdt_(O)_ and T_A(O)_ temperatures.

Regarding the determination of the required thermal regeneration temperature (which, by definition, should be performed in an oxygen atmosphere), it was also found that the amount of the residual non-combustible binder influenced the extent of the binder’s burning in the case of alkali-phenolic binders. The greater the residual inorganic amount of the binder that remained, the higher the required regeneration temperature, and the burning of the organic part was over a wider temperature range (the difference between T_b_ and T_s_).

The next step in the analysis of the alkali-phenolic binders in terms of their thermal regeneration potential was to perform weight loss (WL) and loss on ignition (LOI) tests on the moulding sands that were prepared based on the analysed binders.

**Figure 9:**
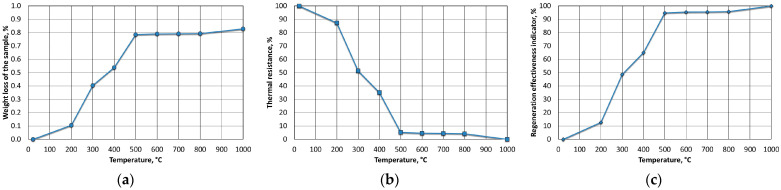
Influence of roasting temperature of spent MSA1 moulding sand on parameters of regeneration process parameters: (**a**) weight loss (WL); (**b**) thermal resistance (R_T_); (**c**) efficiency of thermal regeneration process (W_ETR_).

Figure 9 shows the results for the mass of MSA1, analysing the weight loss (WL) as a function of its annealing temperature in an oven from 200 °C to 800 °C in increments for 1 h (Figure 9a). The samples were not stirred (flipped) or blown with air, creating conditions that were worse than those of standard thermal regeneration equipment. This mode was adopted in order to create more difficult thermal regeneration conditions. Thermal resistance index (R_T_) (Figure 9b) and thermal regeneration process efficiency (W_ETR_) (Figure 9c) were calculated based on the results of weight loss (WL) and determined loss of ignition (LOI).

**Figure 10:**
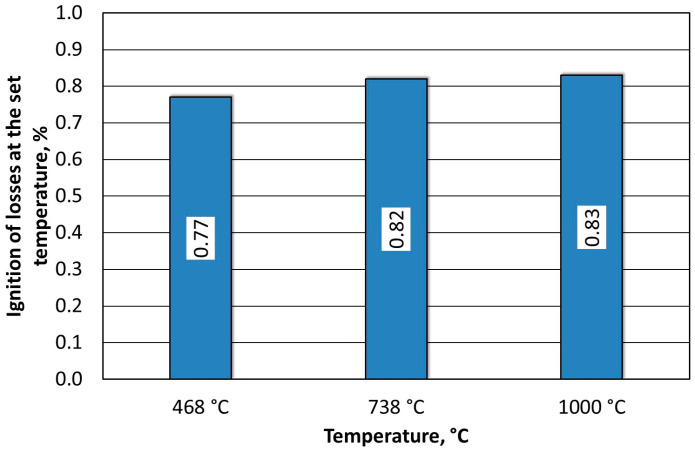
Comparison of weight losses at designated T_s_ and T_b_ temperatures and loss of ignition of spent MSA1 moulding sand.

The spent MSA1 moulding sand that was annealed at increased temperatures showed increasing losses up to 600 °C; both indices stabilised their values from this temperature upwards. A weight-loss test was also performed for the T_s_ and T_b_ temperatures that were determined from the thermogravimetric analysis by roasting them at the same time as the determination of the loss of ignition (2 h). These results are shown in Figure 10.

**Figure 11:**
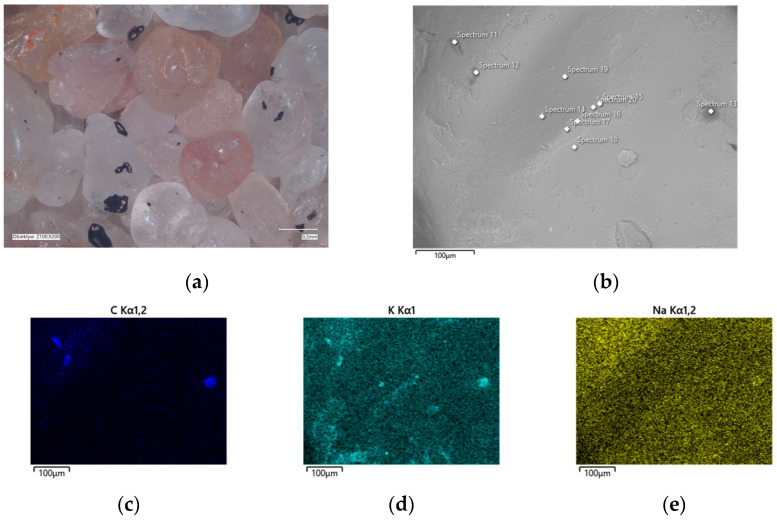
Grain surface morphology of MSA1 moulding sand after thermal regeneration at 468 °C: (**a**) confocal microscope image, mag. ×200; (**b**) SEM image with marked chemical analysis points, mag. ×1000; (**c**) carbon distribution in analysed area; (**d**) potassium distribution in analysed area; (**e**) sodium distribution in analysed area.

**Figure 12:**
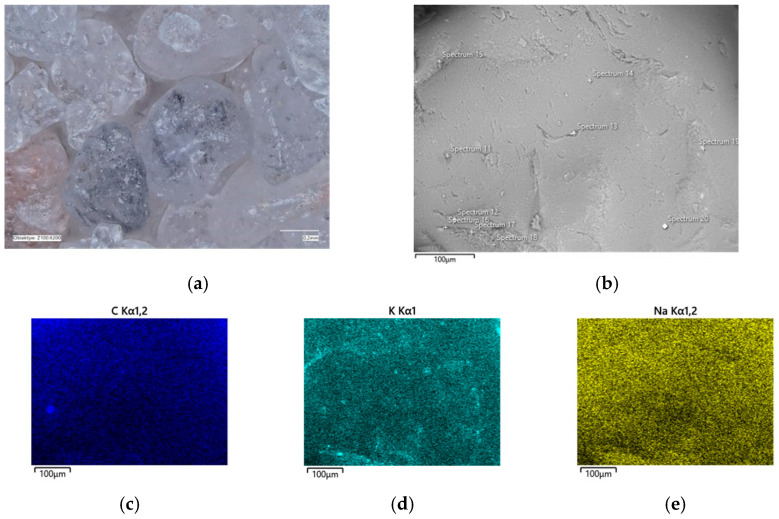
Grain surface morphology of MSA1 moulding sand after thermal regeneration at 738 °C: (**a**) confocal microscope image, mag. ×200; (**b**) SEM image with marked chemical analysis points, mag. ×1000; (**c**) carbon distribution in analysed area; (**d**) potassium distribution in analysed area; (**e**) sodium distribution in the analysed area.

In the determined range of temperatures, the weight losses varied by 0.05%. A confocal microscope image was also taken of the samples after annealing at the designated temperatures (Figure 11a and Figure 12a). As can be seen in Figure 11a, small amounts of the organic part of the binder are visible in the grains; after roasting at a higher temperature, these were no longer found on the samples. SEM images of the tested material samples were also taken (Figure 11b and Figure 12b).

A chemical analysis was also carried out on their surfaces, which is presented in Table 5 and Table 6. The chemical analysis showed a slightly higher amount of carbon at a lower temperature (T_s_). In both samples, the presence of potassium and about ten times less sodium than potassium were clearly visible, indicating that the binder was obtained by reaction with a potassium base. The locations of the concentrations of the carbon, potassium, and sodium elements are also shown as decomposition maps in Figure 11 and Figure 12.

**Figure 13:**
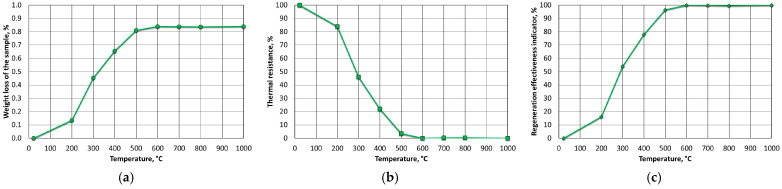
Influence of roasting temperature of spent MSA2 moulding sand on parameters of regeneration process parameters: (**a**) weight loss (WL), (**b**) thermal resistance (R_T_), (**c**) efficiency of thermal regeneration process (W_ETR_).

**Figure 14:**
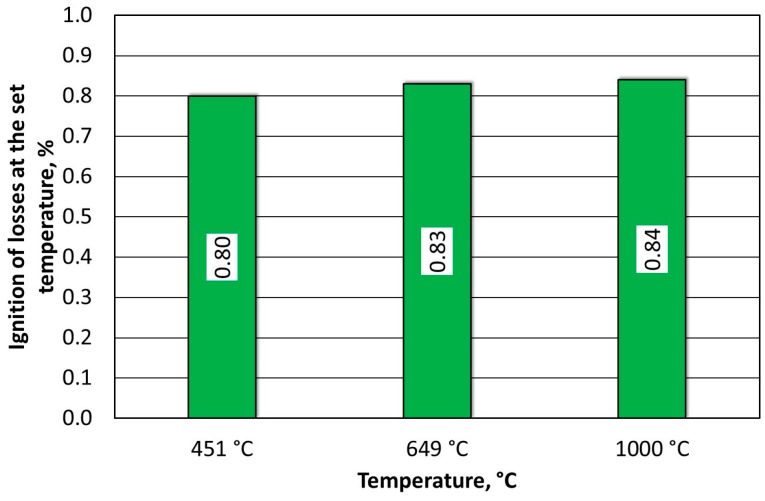
Comparison of weight losses at designated T_s_ and T_b_ temperatures and loss of ignition of spent MSA2 moulding sand.

The test results for the MSA2 moulding sand are shown in Figure 13 For this binder, the weight losses (WLs) were significant up to a roasting temperature of 500 °C; from 600 °C upwards, these was no longer affected. The temperature resistance index as well as the regeneration efficiency index indicated a temperature of 600 °C that was needed to remove the organic part of the binder. For the A2 binder, the onsets of burning temperature T_s_ and burning temperature T_b_ were determined by roasting the binder for 2 h at these temperatures. Within the range of these temperatures, a change of 0.03% was found (as shown in Figure 14).

**Figure 15:**
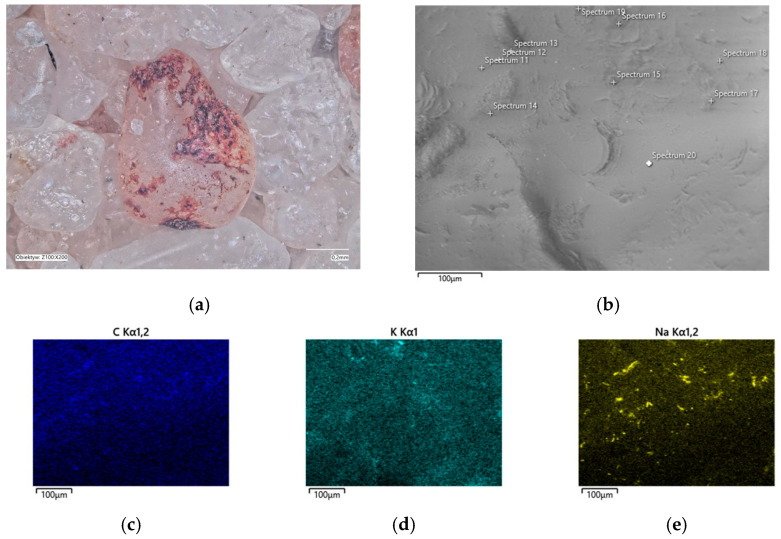
Grain surface morphology of MSA2 moulding sand after thermal regeneration at 451 °C: (**a**) confocal microscope image, mag. ×200; (**b**) SEM image with marked chemical analysis points, mag. ×1000; (**c**) carbon distribution in analysed area; (**d**) potassium distribution in analysed area; (**e**) sodium distribution in analysed area.

**Figure 16:**
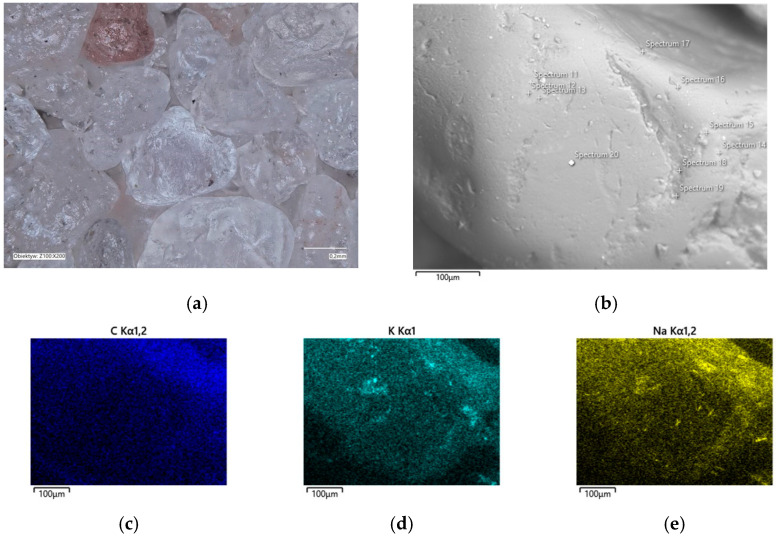
Grain surface morphology of MSA2 moulding sand after thermal regeneration at 649 °C: (**a**) confocal microscope image, mag. ×200; (**b**) SEM image with marked chemical analysis points, mag. ×1000; (**c**) carbon distribution in analysed area; (**d**) potassium distribution in analysed area; (**e**) sodium distribution in analysed area.

The chemical analysis that was performed on the surface of the matrix grains (Figure 15b and Figure 16b) after roasting at the designated temperatures was inconclusive as to which hydroxide was used to prepare the A2 binder; the corresponding results are shown in Table 7 and Table 8. On the contrary, the chemical analysis that was performed on the surfaces of the roasted grains at the designated T_b_ = 649 °C temperature showed higher amounts of total Na and K than at T_s_ = 451 °C; this indicated the changing proportions of the organic and inorganic residues on the grain surfaces. In the confocal microscope image in Figure 15a, trace black spots can be seen, these indicated the presence of incompletely burnt organic matter. In contrast, the matrix grains are bright and free of organic contamination (Figure 16a). The locations of the accumulations of the main binder elements are shown in the distribution maps in Figure 15c–e and Figure 16c–e.

**Figure 17:**
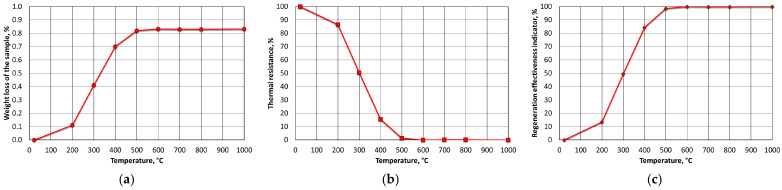
Influence of roasting temperature of spent MSA3 moulding sand on parameters of regeneration process parameters: (**a**) weight loss (WL), (**b**) thermal resistance (R_T_), (**c**) efficiency of thermal regeneration process (W_ETR_).

**Figure 18:**
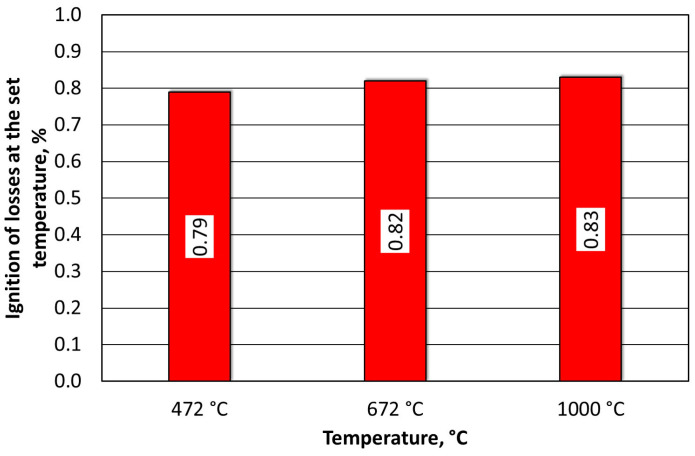
Comparison of weight losses at designated T_s_ and T_b_ temperatures and loss of ignition of spent MSA3 moulding sand.

**Figure 19:**
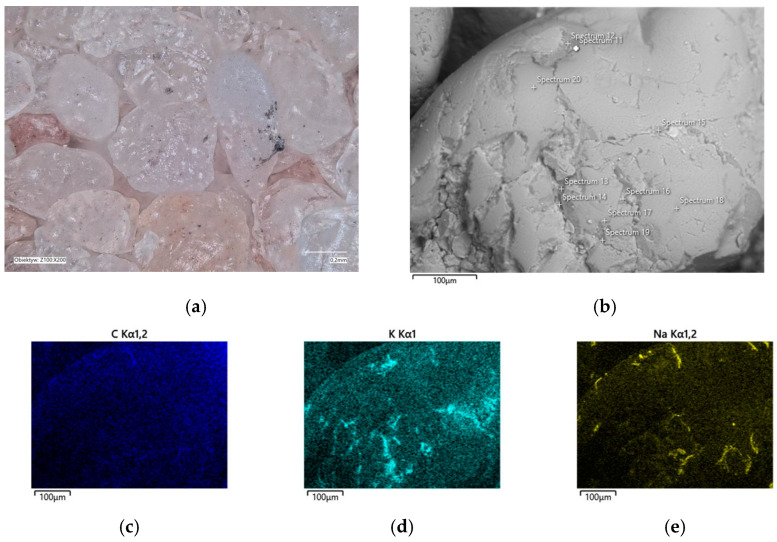
Grain surface morphology of MSA3 moulding sand after thermal regeneration at 472 °C: (**a**) confocal microscope image, mag. ×200; (**b**) SEM image with marked chemical analysis points, mag. ×1000; (**c**) carbon distribution in analysed area; (**d**) potassium distribution in analysed area; (**e**) sodium distribution in analysed area.

**Figure 20:**
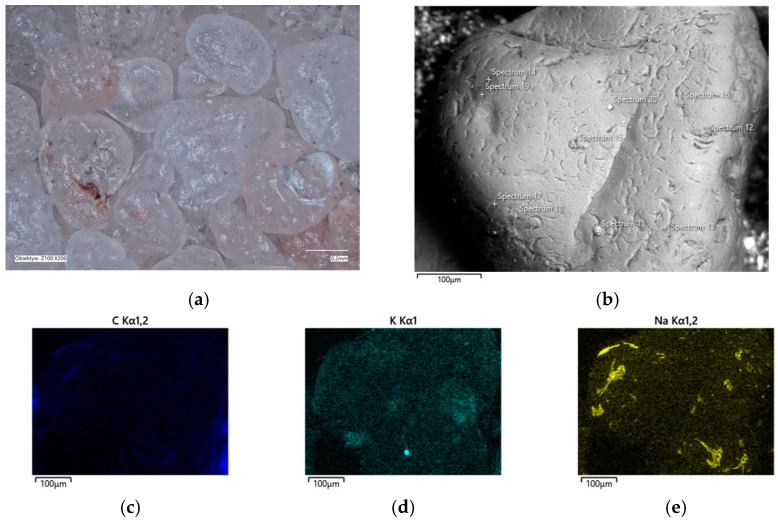
Grain surface morphology of MSA3 moulding sand after thermal regeneration at 672 °C: (**a**) confocal microscope image, mag. ×200; (**b**) SEM image with marked chemical analysis points, mag. ×1000; (**c**) carbon distribution in analysed area; (**d**) potassium distribution in analysed area; (**e**) sodium distribution in analysed area.

Thermal regeneration tests were also carried out on the MSA3 moulding sand. For this moulding sand, the weight losses (WLs) varied significantly up to a temperature of 400 °C; they were less intense up to 500 °C and unchanged from 600 °C upwards. The corresponding results are shown in Figure 17. The ignition losses that were carried out at the designated T_s_ and T_b_ temperatures showed a change in value of 0.03% (Figure 18). Figure 19a and Figure 20a show no significant differences in the surfaces of the matrix grains. The results of the chemical analysis that was performed on the surfaces of the grains after roasting at the determined onset temperature T_s_ (Figure 19b) and the burning temperature T_b_ (Figure 20b) are presented in Table 9 and Table 10. Again, both sodium (Na) and potassium (K) could be found, thus indicating the use of both hydroxides in the preparation of the A3 binder. Their respective distributions on the surfaces of the matrix grains are shown as maps in Figure 19d,e and Figure 20d,e.

## 4. Discussion

**Figure 21:**
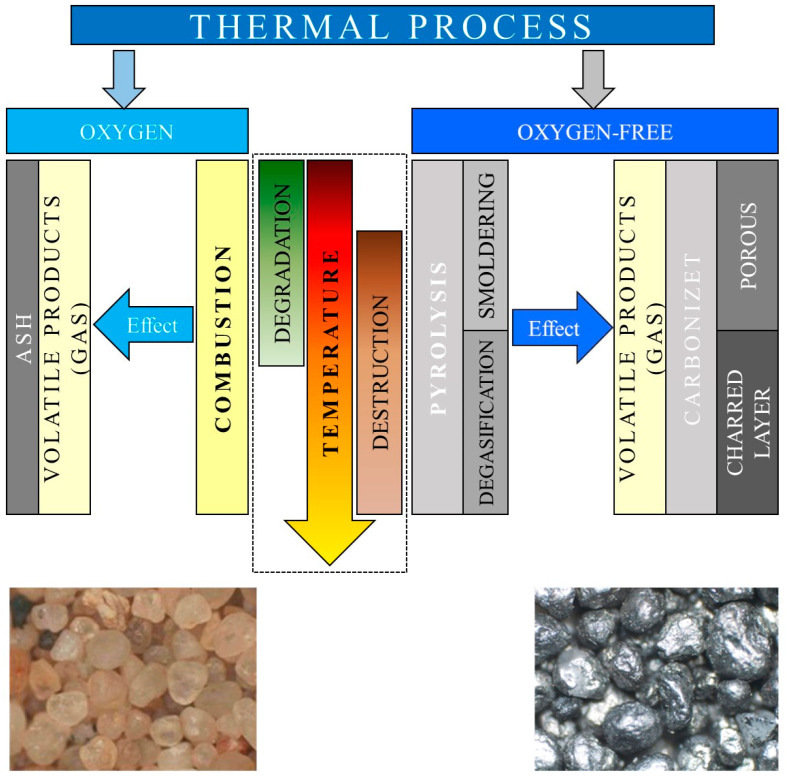
Process diagram for the thermal treatment of waste (particularly spent moulding or cored sand).

The thermal process that was considered as a procedure for the disposal of various types of waste (including spent moulding and core sand) is schematically illustrated in Figure 21.

By supplying heat to moulding sand through a thermal process, the bound organic binder reaches a temperature at which the weakest chemical bonds in the chain begin to break down. This phenomenon is called thermal degradation [63]. For both synthetic polymers and biopolymers, the term “degradation” refers to the deterioration in the functionality of the polymer material and the changes in its physical properties that are caused by the chemical reactions.

Because of the ways in which the process can be initiated, the classification also distinguishes between mechanical, photochemical, radiation, biological, and chemical degradation in addition to thermal degradation, as mentioned above. Thermal degradation occurs when a polymer changes its physical properties under the influence of elevated temperatures. This process occurs without chemical agents. Polymeric materials are rarely chemically pure and usually contain additional components such as dyes, fillers, stabilisers, catalysts, hardeners, or impurities. These additional components in a polymeric material can react with the polymer at elevated temperatures. As a result, it is difficult to distinguish between thermal degradation and so-called thermochemical degradation. The occurrence of thermal degradation in polymeric materials in the foundry industry can be observed when determining the “hot distortion” parameter. This factor allows for a simulation of the behaviour of the finished cores during heating. As presented in [25,64], the fittings lost their initial physical properties and deformed with a change in temperature as a result of the thermal degradation of the binder (depending on the furfuryl alcohol content of the urea–furfuryl resins that was used). For the resin with the lowest amount of furfuryl alcohol in its composition, the bonding properties deteriorated (the binder degraded) at a temperature of about 240 °C.

In general, the thermal degradation of polymers (casting resins) can be stated to occur during processing and usage at elevated temperatures, thus causing the following changes [65]:

- Change in chemical structure of a polymer as a result of chain scission reactions and oxidation processes;
- Change in the average molar mass;
- Cross-linking of the structure;
- Change in physicochemical and mechanical properties;
- Change in the shape and colour of the sample.

It should also be emphasised that, the higher the interaction temperature with the polymer and the higher the heating rate [65],

- the higher rate of thermal decomposition of polymers;
- the more low-molecular decomposition products formed;
- the faster the ignition and flaming of the polymer occurs.

At sufficiently high temperatures, there is an intensive breaking of the chemical bonds in the macromolecules. Most of the bonds break. The process grows exponentially and leads to the destruction (pyrolysis) of the polymer (binder). The products of pyrolysis can be monomers, oligomers, gaseous volatiles, and carbonised materials in the solid state (coke). The results from [63] indicated that the formation of the carbonised layer in the destruction process took place in two stages. When heated to approximately 550 °C, a porous carbonised layer and volatile combustible products were formed, which may have been accompanied by a mist that was formed from the tar particles. At temperatures above 550 °C, the previously formed coke was transformed into a carbonised layer that was composed of randomly distributed particles in a graphite-like structure (independent of the type of starting polymer).

The mechanism of the destruction process and the chemical composition of the products depended on the chemical structure of the macromolecule, the heating rate, the final temperature, and the thermal effects of the reactions that took place (which were endothermic reactions that required energy to proceed). The temperature distribution of the degradation and destruction depended on the valence bond energy. Since this energy varies over a wide range (as shown in [63]), the boundary between the destruction and degradation was therefore blurred; so, both processes could have occurred together.

## 5. Conclusions

The tests that were carried out on spent alkali-phenolic binder moulding sands that were subjected to thermal treatment have allowed some conclusions to be drawn.

Based on the thermogravimetric analyses that were carried out, it was found that the integral decomposition temperature of ipdt and ipdt_(O)_ depended on the contents of the inorganic components that remained after burning at 900 °C. The more inorganic compounds there were in the binder, the higher the determined temperatures were. This meant the greater thermostability of the alkali-phenolic resin in question.

A thermogravimetric analysis of the alkali-phenolic binders that were realised in an oxygen atmosphere was characterised by a periodic section with reduced binder destruction kinetics. This was a feature that affected the burning time of the organic part of the alkali phenolic resins. This was an advantageous property of these binders that was discernible in steel casting, which indicated that the gradual degradation of the alkali-phenolic resin during the first period of the temperature exposure exhibited thermoplastic properties, thus compensating for the thermal expansion of the grain matrix when the mould was flooded with liquid metal; this eliminated any mould cracking and possible liquid metal flow.

The higher proportion of inorganic components in the resin also affected the determination of the required thermal regeneration temperature. The determined burning temperature of the binder (T_b_) was higher. Also, the binder destruction process itself was carried out over a wider temperature range (from the start temperature of T_s_ to this burning temperature T_b_). The less inorganic material in the composition of the alkali-phenolic resin, the smaller the range.

Depending on the composition of the alkali-phenolic resin, the thermal regeneration process also depends on the number of inorganic compounds; this was reflected in the amounts of mass loss, thermal resistance, and efficiency of the regeneration process. The lower number of inorganic compounds that formed the binder resulted in a maximum weight loss at a lower temperature, thus resulting in higher regeneration efficiency (however, the thermal resistance was then weaker). Greater thermal resistance would be obtained for higher amounts of inorganic compounds in the resin due to the signalled slower burning.

The presence of an alkaline catalyst in the alkali-phenolic resins became apparent in the form of sodium and potassium residues on the surface of the matrix grains (particularly in the surface cavities}.

The tests that were carried out on a group of alkali-phenolic binders indicated that a temperature of 500 °C was required for the thermal regeneration of spent moulding sand. However, the longer the regeneration time, the more alkali that was present in the binder that was used. It should also be noted that the thermal process did not remove any inorganic compounds. Mechanical regeneration may have also been ineffective due to the accumulation of sodium and potassium in the surface irregularities of the matrix grains. A full purification would have required intense mechanical action on the matrix (abrasion); however, this would have reduced the grain matrix yield after the regeneration process.

## Figures and Tables

**Table 1:** Materials used in the study.

Identification of the Binder	Resin Name	Name of Hardener	Hardener Ratio (Resin wt.%)
A1	Estrofen Prec-Odlew (Kraków, Poland)	PR Prec-Odlew (Kraków, Poland)	25
A2	Fenotec 280ES Foseco International Limited (Tamworth, UK)	Fenotec HC (Esters (HC30)) Foseco International Limited (Tamworth, UK)	25
A3	Permabind 440 Eurotec (Hagen, Germany)	Permabind Plus 7 Hardener Eurotec (Hagen, Germany)	25

**Table 2:** Summary of calculated parameters for determining thermostability of polymers for binders subjected to thermal analysis in oxygen-free atmosphere.

Parameter	Binder		
	A1	A2	A3
Surface area ABDE	55,881.54	49,220.90	51,117.76
Surface area ABCE	87,500.00	87,500.00	87,500.00
A*	0.638646	0.562525	0.584203
T_A_ (°C)	583.82	517.21	536.18
Surface area FBGH	34,407.39	30,603.92	30,739.89
Surface area HFBN	20,266.36	14,801.82	15,381.52
K*	0.589012	0.4836576	0.500377
A*K*	0.37617	0.272069	0.292322
ipdt (°C)	354.15	263.06	280.78

**Table 3:** Summary of calculated parameters for determining thermostability of polymers for binders subjected to thermal analysis oxygen atmosphere.

Parameter	Binder		
	A1	A2	A3
Surface area ABDE	49,027.39	37,536.83	41,726.82
Surface area ABCE	87,500.00	87,500.00	87,500.00
A*_(O)_	0.560427	0.428992	0.476878
T_A(O)_ (°C)	515.37	400.37	442.27
Surface area FBGH	40,405.86	35,532.26	38,579.30
Surface area HFBN	27,035.83	23,565.58	27,090.40
K*_(O)_	0.669107	0.663217	0.7022
A*_(O)_K*_(O)_	0.374986	0.284515	0.334864
ipdt_(O)_ (°C)	353.11	273.95	318.01

**Table 4:** Parameters determined for tested alkali-phenolic binders.

Binder	Residue after Degradation (%)	ipdt (°C)	T_A_ (°C)	Residue after Destruction (%)	ipdt_(O)_ (°C)	T_A(O)_ (°C)	T_s_ (°C)	T_b_ (°C)
A1	38.43	354	584	17.60	353	515	468	738
A2	37.82	263	517	5.34	274	400	451	649
A3	39.86	281	536	7.54	318	442	472	672

**Table 5:** Elemental wt.% content of spent MSA1 sand after roasting for 2 h at 468 °C in analysed area (Figure 11b).

Element wt.%	C	O	Na	Al	Si	K
Max	37.41	46.62	0.53	2.90	45.24	4.09
Min	7.01	35.71	0.19	0.19	19.83	0.98
Average	16.13	43.08	0.34	0.69	37.37	2.39
Standard Deviation σ	13.30	3.73	0.12	0.83	9.58	1.00

**Table 6:** Elemental wt.% content of spent MSA1 sand after roasting for 2 h at 738 °C in analysed area (Figure 12b).

Element wt.%	C	O	Na	Al	Si	K
Max	25.25	51.80	0.59	3.99	45.35	5.85
Min	6.24	44.73	0.12	0.21	27.79	1.15
Average	10.00	48.04	0.30	1.01	37.64	3.02
Standard Deviation σ	5.59	2.28	0.15	1.16	5.09	1.56

**Table 7:** Elemental wt.% content of spent MSA2 sand after roasting for 2 h at 451 °C in analysed area (Figure 15b).

Element wt.%	C	O	Na	Al	Si	K
Max	13.24	54.22	7.62	0.94	43.88	1.48
Min	6.18	47.87	0.39	0.49	27.12	0.49
Average	8.93	51.64	3.21	0.67	34.71	0.84
Standard Deviation σ	2.60	1.88	2.82	0.17	5.45	0.35

**Table 8:** Elemental wt.% content of spent MSA2 sand after roasting for 2 h at 649 °C in analysed area (Figure 16b).

Element wt.%	C	O	Na	Al	Si	K
Max	9.20	52.63	5.64	4.32	47.22	8.27
Min	5.32	42.84	0.57	0.56	25.79	0.67
Average	7.23	46.69	1.95	1.15	39.87	3.11
Standard Deviation σ	1.37	3.01	1.44	1.15	6.15	2.31

**Table 9:** Elemental wt.% content of spent MSA3 sand after roasting for 2 h at 472 °C in analysed area (Figure 19b).

Element wt.%	C	O	Na	Al	Si	K
Max	10.75	53.52	4.01	5.43	41.42	4.15
Min	6.43	46.99	0.41	0.25	29.28	0.47
Average	7.60	49.71	1.64	2.72	35.79	2.53
Standard Deviation σ	1.30	2.18	0.92	1.90	3.39	1.51

**Table 10:** Elemental wt.% content of spent MSA3 sand after roasting for 2 h at 672 °C in analysed area (Figure 20b).

Element wt.%	C	O	Na	Al	Si	K
Max	11.53	51.66	5.88	1.19	44.42	10.23
Min	5.94	45.44	0.55	0.24	26.34	0.43
Average	8.42	49.67	2.44	0.54	36.96	1.97
Standard Deviation σ	1.83	2.32	1.93	0.32	5.68	2.98

## Data Availability

The data that support the findings of this study are available from the author (M.Ł.) upon reasonable request.
